# Exploring the pediatric nasopharyngeal bacterial microbiota with culture-based MALDI-TOF mass spectrometry and targeted metagenomic sequencing

**DOI:** 10.1128/mbio.00784-24

**Published:** 2024-04-29

**Authors:** Sreymom Pol, Teemu Kallonen, Tommi Mäklin, Poda Sar, Jill Hopkins, Sona Soeng, Thyl Miliya, Clare L. Ling, Stephen D. Bentley, Jukka Corander, Paul Turner

**Affiliations:** 1 Cambodia Oxford Medical Research Unit, Angkor Hospital for Children, Siem Reap, Cambodia; 2 Department of Biostatistics, University of Oslo, Oslo, Norway; 3 Wellcome Sanger Institute, Hinxton, United Kingdom; 4 Department of Mathematics and Statistics, University of Helsinki, Helsinki, Finland; 5 Nuffield Department of Medicine, Centre for Tropical Medicine and Global Health, University of Oxford, Oxford, United Kingdom; 6 Helsinki Institute for Information Technology HIIT, Department of Mathematics and Statistics, University of Helsinki, Helsinki, Finland; The University of Kansas Medical Center, Kansas City, Kansas, USA

**Keywords:** colonization, pediatrics, Cambodia, *Streptococcus pneumoniae*, *Haemophilus influenzae*, *Staphylococcus aureus*, MALDI-TOF, metagenomic sequencing

## Abstract

The nasopharynx is an important reservoir of disease-associated and antimicrobial-resistant bacterial species. This proof-of-concept study assessed the utility of a combined culture, matrix-assisted laser desorption/ionization-time of flight mass spectrometry (MALDI-TOF MS), and targeted metagenomic sequencing workflow for the study of the pediatric nasopharyngeal bacterial microbiota. Nasopharyngeal swabs and clinical metadata were collected from Cambodian children during a hospital outpatient visit and then biweekly for 12 weeks. Swabs were cultured on chocolate and blood-gentamicin agar, and all colony morphotypes were identified by MALDI-TOF MS. Metagenomic sequencing was done on a scrape of all colonies from a chocolate agar culture and processed using the mSWEEP pipeline. One hundred one children were enrolled, yielding 620 swabs. MALDI-TOF MS identified 106 bacterial species/40 genera: 20 species accounted for 88.5% (2,190/2,474) of isolates. Colonization by *Moraxella catarrhalis* (92.1% of children on ≥1 swab), *Haemophilus influenzae* (87.1%), and *Streptococcus pneumoniae* (83.2%) was particularly common. In *S. pneumoniae*-colonized children, a median of two serotypes [inter-quartile range (IQR) 1–2, range 1–4] was detected. For the 21 bacterial species included in the mSWEEP database and identifiable by MALDI-TOF, detection by culture + MALDI-TOF MS and culture + mSWEEP was highly concordant with a median species-level agreement of 96.9% (IQR 86.8%–98.8%). mSWEEP revealed highly dynamic lineage-level colonization patterns for *S. pneumoniae* which were quite different to those for *S. aureus*. A combined culture, MALDI-TOF MS, targeted metagenomic sequencing approach for the exploration of the young child nasopharyngeal microbiome was technically feasible, and each component yielded complementary data.

**IMPORTANCE:**

The human upper respiratory tract is an important source of disease-causing and antibiotic-resistant bacteria. However, understanding the interactions and stability of these bacterial populations is technically challenging. We used a combination of approaches to determine colonization patterns over a 3-month period in 101 Cambodian children. The combined approach was feasible to implement, and each component gave complementary data to enable a better understanding of the complex patterns of bacterial colonization.

## INTRODUCTION

The human infant nasopharynx is an important site of colonization for disease-associated commensal bacteria, including *Haemophilus influenzae*, *Moraxella catarrhalis*, *Staphylococcus aureus*, and *Streptococcus pneumoniae*. Colonization is highly dynamic and develops with age (1–3). The infant bacterial microbiota is influenced by the mode of delivery (4), breastfeeding (5), socio-economic status (6), ethnicity (7), innate immunity receptor gene polymorphisms (8), and geolocation (9). Bacterial colonization may also follow seasonal patterns and be impacted by viral co-infection (10–12). Differences in microbiota profiles may have impact on the susceptibility to respiratory tract infections (3).

Nasopharyngeal colonizers are an important reservoir of antimicrobial resistance (AMR). Antibiotic administration for acute otitis media reduces colonization by antimicrobial-susceptible organisms (13, 14). AMR may also increase in nasopharyngeal organisms because of exposure to antibiotics used for prophylaxis or treatment of infections in a distant site (15). For example, prophylaxis to prevent *Pneumocystis jirovecii* infection in HIV-positive infants results in colonization by co-trimoxazole-resistant pneumococci (16).

Studies of the nasopharyngeal microbiota have historically been culture based with a focus on a limited range of organisms with pathogenic potential (17–19). Expanding species detection based on multiple colony picks and conventional bacteriological techniques is possible but labor intensive (20). Culture-based microbiota studies may be improved by the application of matrix-assisted laser desorption/ionization-time of flight mass spectrometry (MALDI-TOF MS) for bacterial identification. MALDI-TOF MS systems are now commonplace in diagnostic microbiology laboratories and are capable of high-throughput, cost-effective, accurate, and rapid identification of a broad range of bacterial and fungal species (21). A small study of broncho-alveolar lavage fluid comparing culture followed by MALDI-TOF MS against 16S sequencing direct from the specimen revealed reasonable concordance, with mostly fastidious anaerobes being missed by culture (e.g., *Prevotella* sp.) and some readily culturable organisms being missed by sequencing (22).

In recent years, molecular approaches, i.e., amplicon sequencing of the 16S rRNA gene or full metagenomic sequencing, have become more commonplace. However, the low biomass of the nasopharynx and associated samples renders this approach challenging (23, 24). Successful sequence-based studies from Australia (1), the Netherlands (11), and Thailand (25) have all revealed a fairly small number of dominant taxa.

Targeted metagenomics, defined here as where specimens are cultured and DNA from culture plates is extracted and sequenced, is a potentially important and cost-effective way to improve resolution of sequencing for key species. It has been applied recently to determine pneumococcal colonization diversity in a cohort of mother-infant pairs on the Thailand-Myanmar border (26), and changes in the pneumococcal resistome over time in South African infants (27).

In this proof-of-concept study, a combined culture-based workflow using MALDI-TOF MS and targeted metagenomic sequencing was assessed using a collection of nasopharyngeal swabs (NPS) from a cohort of Cambodian children presenting to a hospital out-patient department with minor illnesses. Colonization by major lineages of *H. influenzae*, *M. catarrhalis*, *S. aureus*, and *S. pneumoniae* was explored in greater detail using the recently described, and well benchmarked, mSWEEP pipeline (28).

## MATERIALS AND METHODS

### Study population

Children aged 5 months to 4 years were recruited from the Angkor Hospital for Children (AHC) out-patient department. Children were eligible for study enrolment if they presented to the hospital with a minor illness not requiring hospital admission and had not knowingly received a systemic antibiotic in the preceding 4 weeks. The sample size was not calculated formally for this proof-of-concept study, conducted in a population with a known high prevalence of pneumococcal colonization (29, 30). Recruitment was purposive, aiming to capture 25 children who were prescribed amoxicillin during their out-patient visit and 75 children who were not prescribed an antibiotic.

Angkor Hospital for Children is a non-governmental healthcare organization located in Siem Reap, northern Cambodia (31). The hospital provides free primary- to tertiary-level care to children <16 years old without geographic restrictions. AHC has 82 beds, with 117,232 out-patient visits and 3,189 admissions recorded in 2018. Cambodia is a lower middle-income South East Asian country, with a tropical climate. In 2018, the under-five mortality was 27.7/1,000 live births (32). The *H. influenzae* type b (Hib) vaccine was introduced in 2010 and the 13-valent pneumococcal conjugate vaccine (PCV13) followed in 2015 (33). Vaccine coverage was 92% (Hib) and 84% (PCV13) among one-year olds in 2018 (34).

### Study procedures

At the enrolment visit, demographic, immunization, and current illness data were recorded and a flocked nylon NPS was collected (Medical Wire & Equipment, Corsham, UK). Children were followed up at six time points, at 2-week intervals, until 12 weeks post-enrolment. At each follow-up visit, details of recent illness and medications were recorded, vital signs were taken, and a further NPS was collected.

NPS were placed immediately into 1 mL sterile skim milk-tryptone-glucose-glycerol broth (STGG) and kept in a cool box before vortex mixing and separation into two 0.5-mL aliquots which were stored at −80°C within 8 hours of collection (35). Onsite laboratory processing is summarized in Fig. S1.

### Culture-based detection of colonization

NPS-STGG specimens (swab aliquot #1, containing the swab tip) were thawed, and 10 µL was cultured onto chocolate agar (CA) and 5% sheep blood agar + 5 mg/L gentamicin (BA-CN) plates. Growth was assessed after overnight incubation at 37°C in 5% CO_2_. Plates with poor growth were discarded and culture repeated using 100 µL NPS-STGG. Bacteriological media were prepared in-house using locally sourced antibiotic-free citrated sheep blood and commercial reagents (Oxoid, Basingstoke, UK), with the use of appropriate quality controls.

All discrete colony morphotypes from the CA plate were identified by MALDI-TOF MS (VITEK MS, Knowledge Base V3.2.0; bioMerieux, Marcy L’ Etoile, France). The *in vitro* diagnostic (IVD) mode was used primarily, and colonies were re-tested using the research use only (RUO) mode if an acceptable result was not obtained in the IVD mode. An acceptable result was defined as return of a single organism name with an associated confidence level. If >1 organism name from the same genus was returned, then the result was entered to the genus level (e.g., “*Streptococcus* sp.”). Where multiple genera were offered for a given colony pick, attempts were made to purify and retest. In the event of repeated failure to identify by MALDI-TOF MS, then just the Gram result for the colony was recorded (e.g., “Gram positive cocci”).

Specific target species were characterized in greater detail. Beta-lactamase activity was determined for *H. influenzae* isolates using Cefinase disks (BBL, Becton Dickinson, Franklin Lakes, NJ, USA). Methicillin resistance was determined for *S. aureus* isolates by cefoxitin disk diffusion testing (Oxoid), following 2018 Clinical and Laboratory Standards Institute guidelines (36). *S. pneumoniae* were followed up from the selective BA-CN culture plate. Identification of the dominant alpha-hemolytic colony morphotype was confirmed by MALDI-TOF MS and optochin disk susceptibility (Oxoid). The serotype was determined by latex agglutination, with confirmation by the Quellung reaction where required (30). The penicillin minimum inhibitory concentration (MIC) was determined using the Etest method (bioMerieux) with non-susceptible defined as an MIC of ≥0.12 µg/mL (36).

### Detection of viral infections

Enrolment visit swabs were tested by PCR to detect the presence of influenza A, influenza B, and respiratory syncytial virus (RSV). Briefly, RNA was extracted from 200 µL thawed NPS-STGG (swab aliquot #2) using the Qiagen Viral RNA Mini Kit and a QIAcube instrument (Qiagen, Hilden, Germany). Multiplex real-time PCR was done using the Fast-Track Diagnostics FLU/HRSV RUO Kit (Siemens Healthcare, Erlangen, Germany) on a Bio-Rad CFX96 thermocycler (Bio-Rad, Hercules, CA, USA). All extraction and PCR work followed the manufacturer’s instructions.

### Targeted metagenomic sequencing-based detection of colonization

At the same time as the primary culture work, a further 100 µL thawed NPS-STGG (swab aliquot #1) was cultured on chocolate agar at 37°C in 5% CO_2_. Following overnight incubation, all colonies from the plate were scraped into 1 mL sterile phosphate buffered saline and centrifuged at full speed for 5 minutes to yield a cell pellet. Following storage at −80°C, DNA was extracted from the cell pellets using the Promega Wizard Genomic Purification DNA Kit (Promega, Madison, WI, USA), following the manufacturer’s instructions. DNA yield and quality were assessed using a BioPhotometer D30 (Eppendorf, Hamburg, Germany), before shipping to the Wellcome Sanger Institute for sequencing on the Illumina HiSeq4000 platform [150 bp paired-end reads, median 15.8 million reads per sample with inter-quartile range (IQR) 14.6–17.2 million]. Read accession numbers are summarized in Table S1. The sequencing-based analysis was performed blinded without the knowledge of the culture or MALDI-TOF MS-based results. The mSWEEP pipeline (version 1.3.2) was performed in accordance with the instructions at GitHub (https://github.com/PROBIC/mSWEEP). In short, first, a reference database of 5,510 taxa was constructed (Table S2) and indexed with Themisto (version 0.1.0; *k* = 31); then, the reads were pseudoaligned also with Themisto. Lastly, mSWEEP was used to obtain the abundances running it with the alignment and Themisto index. The reference included 91 *Haemophilus influenzae*, 55 *Moraxella catarrhalis*, 2 *Moraxella canis*, 13 *Neisseria meningitidis*, 3,041 *Streptococcus pneumoniae*, 2,239 *Staphylococcus aureus*, 447 other *Streptococcaceae* genomes, and single genomes from *Cutibacterium granulosum*, *Corynebacterium pseudodiphtheriticum*, *Corynebacterium accolens*, *Haemophilus parainfluenzae*, *Neisseria bacilliformis*, *Neisseria cinerea*, *Neisseria dentiae*, *Neisseria elongata* subsp. *glycolytica*, *Neisseria flavescens*, *Neisseria lactamica*, *Neisseria mucosa*, *Neisseria perflava*, *Neisseria polysaccharea*, *Neisseria sicca*, *Neisseria subflava*, *Neisseria weaveri*, “*Candidatus* Ornithobacterium hominis” (37), *Staphylococcus argenteus*, *Staphylococcus epidermidis*, *Staphylococcus haemolyticus*, *Staphylococcus saprophyticus*, and *Staphylococcus schweitzeri*.

### Data management and analysis

Clinical and culture-based laboratory data were recorded on paper forms and single entered into an Access 2016 database (Microsoft, Richmond, WA, USA). Automated checks for missing and out-of-range values were implemented in R (R Foundation for Statistical Computing, Vienna, Austria).

Analyses were done using R v4.2.0 (38), with add-on packages tidyverse v1.3.1 for data visualization (39), pheatmap v1.0.12 for hierarchical clustering (40), and vegan v.2.6-2 for calculation of diversity indices, non-metric multidimensional scaling plots, and PERMANOVA (41). Between-group comparisons of proportion data were done using the chi-squared or Fisher’s exact test, as appropriate. Between-group comparisons of continuous data were done using Student’s *t*-test or Wilcoxon rank sum test, as appropriate.

## RESULTS

### Baseline characteristics and clinical follow-up

Between February and May 2018, 101 children were enrolled in the study: 24 in the amoxicillin-treated group and 77 in the no antibiotic group. The measured temperature was higher in the amoxicillin group compared with the no antibiotic group (median 37.6°C vs 36.7°C, *P* = 5.3 × 10^−5^), and these children were more likely to be given a diagnosis of pneumonia (45.8% vs 1.3%). Other baseline characteristics were similar. Respiratory virus infection was very uncommon, with two influenza B, one influenza A, and no RSV detections (Table 1). At enrollment, 60/101 (59.4%) of children were colonized by *S. pneumoniae* and almost two-thirds of isolates (61.7%, 37/60) were penicillin non-susceptible. *H. influenzae* colonization was also common, found in 64 (63.4%) children, with one-third (34.4%, 22/64) isolates being beta-lactamase positive. *S. aureus* colonization was uncommon (nine children, 8.9%), but almost half of the isolates (44.4%, 4/9) were methicillin resistant.

**TABLE 1:** Baseline demographic, clinical, and colonization characteristics in 101 enrolled children

Variable	All	Amoxicillin treatment	No antibiotic	*P* value
	(*n* = 101)	(*n* = 24)	(*n* = 77)	
Demographic details				
Age in months, median (IQR)	17.5 (10.1–32.6)	15.7 (11.2–31.2)	21.2 (10.1–33.7)	0.6
Weight in kg, median (IQR)	9.4 (8.5–11.5)	9.6 (8.8–11.9)	9.4 (8.5–11.4)	0.7
Height in cm, median (IQR)	78.0 (71.5–86.5)	79.5 (74.0–85.8)	77.0 (71.5–86.5)	1.0
Gender, *n* (female:male)	56:45	10:14	46:31	0.2
PCV immunized^*a*^, *n* (%)	78 (77.2)	19 (79.2)	59 (76.6)	1.0
Hib immunized^*a*^, %	97 (96.0)	24 (100)	73 (94.8)	0.2
Breast milk fed^*b*^, *n* (%)	33 (32.7)	6 (25.0)	27 (35.1)	0.5
Day care attendance, *n* (%)	10 (9.9)	2 (8.3)	8 (10.4)	1.0
Household size, median (IQR)	5 (4–7)	5 (4–7)	5 (4–8)	0.7
Smoker in household, *n* (%)	40 (39.6)	8 (33.3)	32 (41.6)	0.6
Baseline visit				
Respiratory symptoms^*c*^, %	78 (77.2)	21 (87.5)	57 (74.0)	0.3
Temperature in °C, median (IQR)	36.9 (36.6–37.5)	37.6 (37.3–37.9)	36.7 (36.7–37.4)	5.3 × 10^−5^
Respiratory rate /min, median (IQR)	33 (28–38)	36 (32–39)	32 (28–37)	0.06
Clinical diagnosis, *n* (%)				2.5 × 10^−7^
Upper respiratory tract infection	78 (77.2)	13 (54.2)	65 (84.4)	
Bronchiolitis	1 (1.0)	0 (0.0)	1 (1.3)	
Pneumonia	12 (11.9)	11 (45.8)	1 (1.3)	
Other	10 (9.9)	0 (0.0)	10 (13.0)	
Respiratory virus detection, *n* (%)				
Influenza A	1 (1.0)	1 (4.2)	0 (0.0)	0.2
Influenza B	2 (2.0)	1 (4.2)	1 (1.3)	0.4
Respiratory syncytial virus	0 (0.0)	0 (0.0)	0 (0.0)	–^*d*^
Bacterial colonization, *n* (%)				
*Streptococcus pneumoniae*	60 (59.4)	17 (70.8)	43 (55.8)	0.2
Penicillin-non-susceptible isolates	37 (61.7)	11 (64.7)	26 (60.5)	1.0
*Haemophilus influenzae*	64 (63.4)	16 (66.7)	48 (62.3)	0.8
Beta-lactamase-positive isolates	22 (34.4)	5 (31.2)	17 (35.4)	1.0
*Staphylococcus aureus*	9 (8.9)	2 (8.3)	7 (9.1)	1.0
Methicillin-resistant isolates	4 (44.4)	0 (0.0)	4 (5.7)	0.4

^*a*^ Defined as ≥2 doses.

^*b*^ Partial or complete.

^*c*^ Cough, sore throat, runny nose, and ear pain; IQR: inter-quartile range.

^*d*^ ”–”, not applicable.

**Fig 1:**
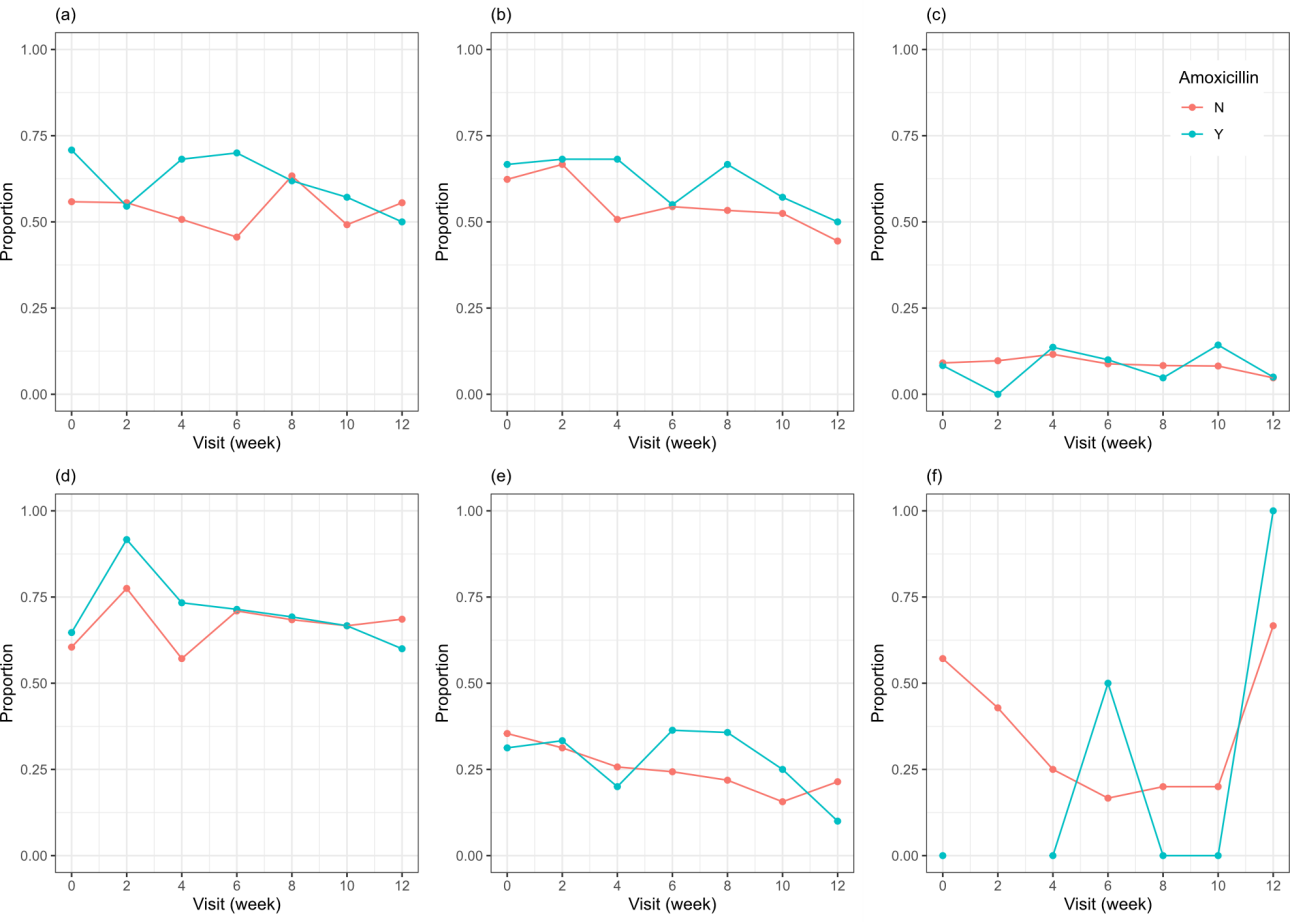
Nasopharyngeal colonization by *Streptococcus pneumoniae*, *Haemophilus influenzae*, and *Staphylococcus aureus* over time in 620 swabs from 101 children sampled up to seven times over 12 weeks. The top row summarizes colonization prevalence by visit for (a) *S. pneumoniae*, (b) *H. influenzae*, and (c) *S. aureus*. The bottom row provides key antimicrobial resistance by species, showing the proportion of (d) *S. pneumoniae* isolates that were penicillin non-susceptible, (e) *H. influenzae* isolates that were beta-lactamase producers (i.e., ampicillin resistant), and (f) *S. aureus* isolates that were methicillin resistant. Turquoise points/lines indicate children in the amoxicillin group, and red points/lines indicate children in the no antibiotic group.

There were 519 follow-up visits (85.6% of expected), yielding a median of seven swabs per child (IQR 6–7, range 1–7) and 620 swabs in total. All seven swabs were collected in 71 (70.3%) children with no difference between amoxicillin and no antibiotic groups (*P* = 0.6). Household respiratory infections occurred frequently between study visits, being documented on 202 occasions (38.9%): 40/126 (31.7%) for the amoxicillin group versus 162/393 (41.2%) for the no antibiotic group (*P* = 0.06). Clearly documented receipt of antibiotics during follow-up was relatively uncommon, with study children receiving an antibiotic on 17 occasions since the preceding visit: 4 (3.2%) in the amoxicillin group and 13 (3.3%) in the no antibiotic group (*P* = 1.0). However, unknown medications were given to study children on 87 occasions during follow-up: 18 (14.3%) in the amoxicillin group and 69 (17.6%) in the no antibiotic group (*P* = 0.8). Colonization by *S. pneumoniae*, *H. influenzae*, and *S. aureus* was relatively stable over time (Fig. 1). There were 345 pneumococcal isolates cultured comprising 32 serotypes (Table S3; Fig. S2): 130 (37.7%) were PCV13 serotypes, 201 (58.3%) were non-vaccine serotypes, and 14 (4.0%) were non-typeable. Eighty-four children were colonized by *S. pneumoniae* on at least one time point; these children were colonized by a median of two serotypes (IQR 1–2, range 1–4).

### Bacterial carriage dynamics assessment using culture and MALDI-TOF MS

**Fig 2:**
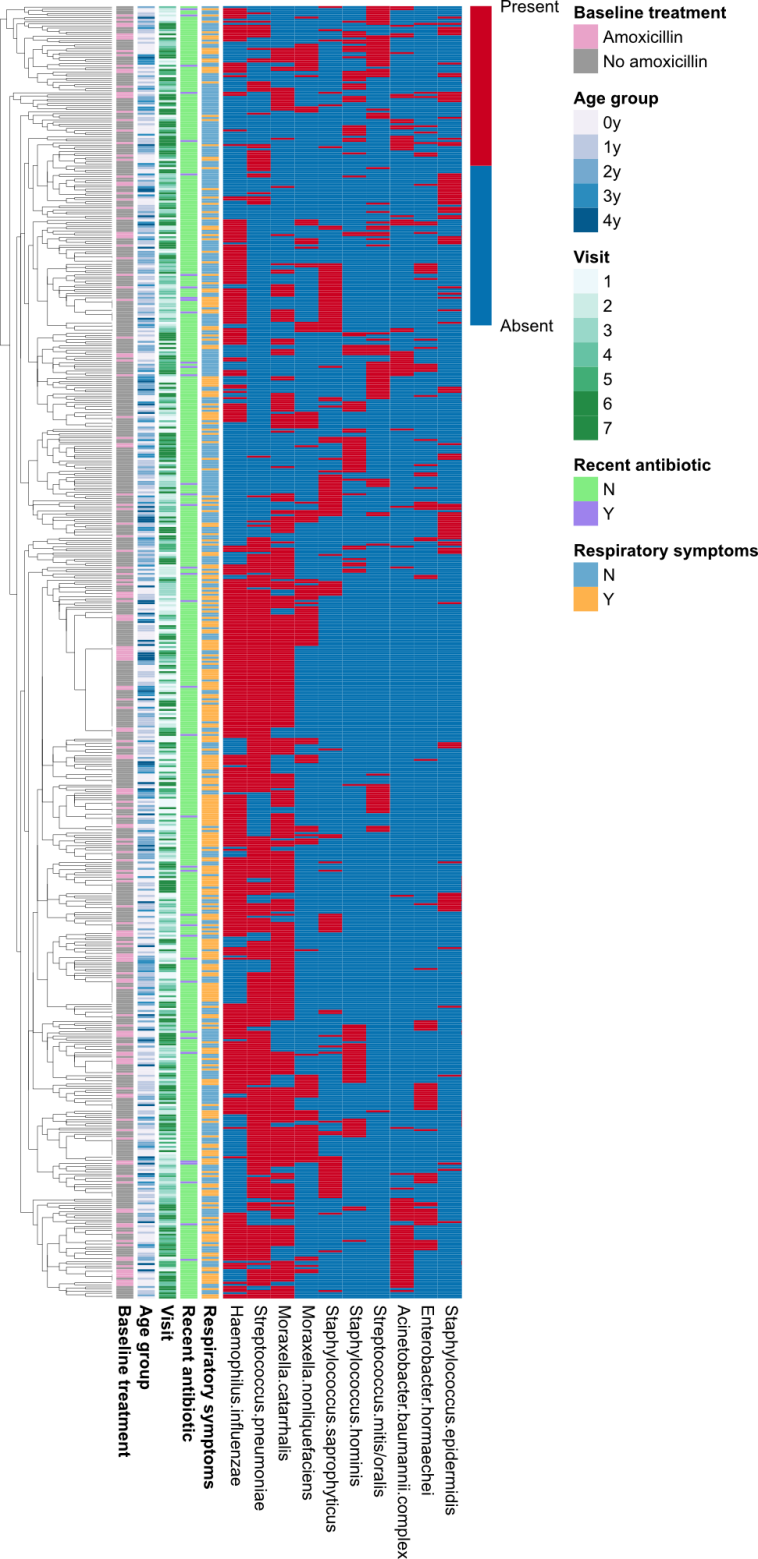
Hierarchical clustering analysis of culture + MALDI-TOF MS data for 618 nasopharyngeal swabs. Each row indicates an individual swab sample, with species represented by columns (red = present, blue = absent). Two outliers were removed from the original data set of 620 swabs. For clarity, only the 10 most frequently identified species are shown. The full figure is included as Fig. S8.

MALDI-TOF MS of CA colony picks identified 106 bacterial species from 40 genera (Fig. S3 and S4), with 20 species accounting for 88.5% (2,190/2,474) of all isolates. Over 40% of the isolates characterized was *M. catarrhalis* (361, 14.6%), *H. influenzae* (353, 14.3%), or *S. pneumoniae* (345, 13.9%). Overall, 92.1% (93/101) of the children was colonized on at least one time point by *M. catarrhalis*, 87.1% (88/101) by *H. influenzae*, 83.2% (84/101) by *S. pneumoniae*, and 29.7% (30/101) by *S. aureus*. Of the 619/620 swabs with detectable growth, the median number of colonies screened was 4 (IQR 3–5; range 1–11), yielding a median of four species (IQR 3–4) and three genera (IQR 3–4) per swab at each time point, with no differences by receipt of amoxicillin at the baseline visit. Alpha diversity did not vary over time, with the median Shannon diversity index ranging from 1.24 to 1.61 and median Simpson diversity index ranging from 0.71 to 0.80 (Fig. S5). Beta-diversity (based on Jaccard distances, given input data of the presence/absence of species) was assessed on the 101 baseline visit swabs, with no clear differences based on age group or clinical diagnosis (Fig. S6 and S7). Exploratory permutational multivariate analysis of variance (PERMANOVA) analysis of the baseline visit swab data did not reveal any correlation between Jaccard distances and environmental or clinical factors (Table S4). Following removal of two extreme outlier swabs (one with no bacterial growth and another which grew only *Corynebacterium* sp. and *Pseudomonas stutzeri*, which resulted in failure of model convergence), hierarchical clustering was performed on the longitudinal swab set (*n* = 618; method: “complete:” distance: “Euclidian”): no obvious associations were found between colonization patterns and child age, baseline amoxicillin treatment, visit number, receipt of an antibiotic since the previous study visit, or current respiratory symptoms (Fig. 2; Fig. S8).

### Comparison of species detection by culture plus MALDI-TOF and culture followed by targeted metagenomic sequencing

**Fig 3:**
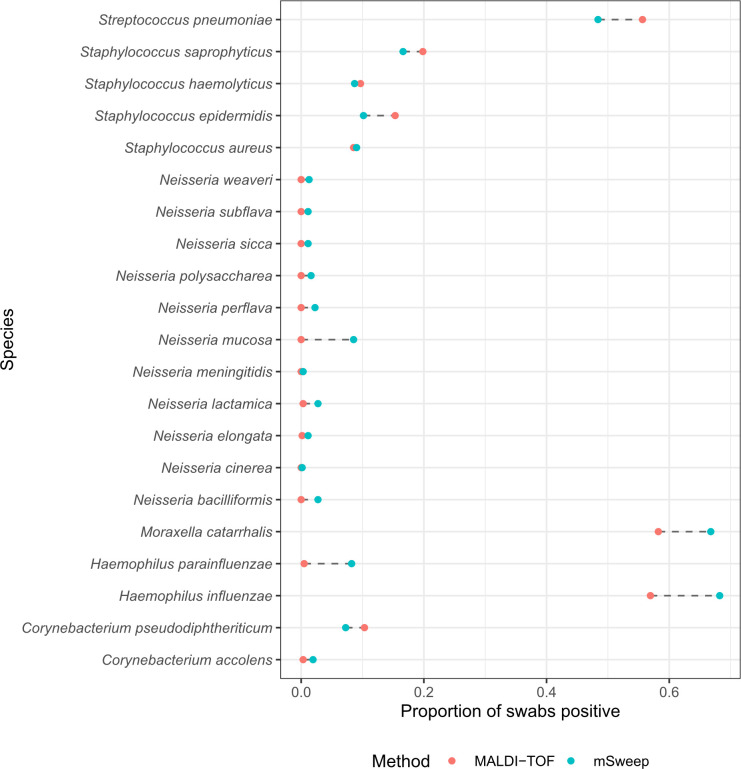
Detection of individual bacterial species by culture + MALDI-TOF MS compared with culture + mSweep in 620 swabs.

All 620 NPS culture CA plate scrapes were sequenced and analyzed using mSWEEP. Comparing the 21 bacterial species included in the mSWEEP database and the VITEK MS knowledgebase, using 1% relative abundance to define presence by sequencing, there was 93.2% detection agreement between culture + MALDI-TOF MS and culture + mSWEEP (Table S5). Species-level comparisons revealed a median agreement between detection methods of 96.9% (IQR 86.8%–98.8%; Fig. 3). *S. pneumoniae* was more frequently detected in NPS by culture + MALDI-TOF MS compared with culture + mSWEEP (55.6% vs 48.4%), with the opposite finding for *H. influenzae* (56.9% vs 68.2%), *M. catarrhalis* (58.2% vs 66.8%), and *S. aureus* (8.6% vs 9.0%, all *P* < 2.2 × 10^−16^). Being negative by culture + MALDI-TOF MS compared with culture + mSWEEP appeared largely due to relative abundance: the median mSWEEP relative abundance was 5.3% (IQR 2.0%–16.9%) for species non-detectable by culture versus 26.8% (IQR 9.9%–62.7%) for those that were culture positive (*P* < 2.2 × 10^−16^; Fig. S9).

### Bacterial carriage dynamics assessment using targeted metagenomic sequencing data

**Fig 4:**
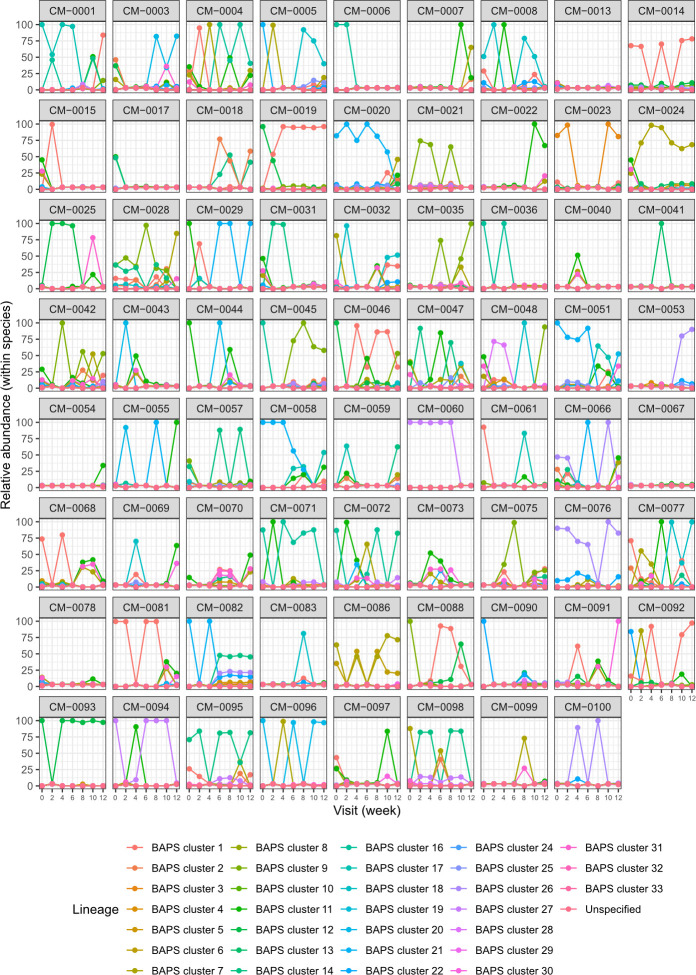
Dynamics of *Streptococcus pneumoniae* colonization over time, in 71 children with complete nasopharyngeal swab sets. Each child is documented in an individual plot, with the within-species relative abundance of each pneumococcal BAPS cluster plotted as a separate color.

Further analysis of mSWEEP data from the 71 children with complete swab sets revealed extensive within-species diversity and temporal instability. As expected from the size and number of regionally relevant genomes in the reference database, almost all the observed diversity was characterized for *S. pneumoniae.* A median of 4 (IQR 2–7) lineages (BAPS clusters) was detected per child, with only two children (CM-0070 and CM-0082) colonized by strains not included in the database (Fig. 4; Table S6). Most children were colonized by several lineages, with variable relative abundance at each time point (e.g., CM-004). Others were stably colonized by a single lineage (CM-0093). A minority of children were non-colonized (e.g., CM-0067). In contrast, *S. aureus* colonization was uncommon, with children being colonized briefly by a single clonal complex (CC) (Fig. S10). However, the apparent detection of multiple *S. aureus* CCs in a child at a single timepoint (e.g., CM-047, visit 3) suggests that the reference database may have been sub-optimal for this species.

## DISCUSSION

This proof-of-concept study has demonstrated the practical utility of a combined culture, MALDI-TOF MS plus targeted metagenomic sequencing approach to the analysis of nasopharyngeal swabs. Data presented highlight the diversity and longitudinal dynamics of the aerobic nasopharyngeal bacterial microbiota in young Cambodian children. Over a 3-month period, most children were colonized by the major respiratory tract pathogens *S. pneumoniae*, *H. influenzae*, and *M. catarrhalis*. The mSWEEP pipeline revealed considerable within-species diversity, which was striking given the short follow-up time.

The aerobic nasopharyngeal bacterial microbiota was dominated by a small number of genera and species. Despite methodologic differences, this finding is similar to previous 16S or full metagenomic sequencing studies. Teo and colleagues identified six dominant genera (*Moraxella* [31.2%], *Streptococcus* [15.5%], *Corynebacterium* [13.5%], *Staphylococcus* [10.3%], *Haemophilus* [9.7%], and *Alloiococcus*/*Dolosigranulum* [8.8%]) in a study of 234 Australian infants over the first year of life (1). A study of 96 Dutch children found great inter-individual variability but with just 30 operational taxonomic units (OTU) representing almost 98% of all sequencing reads (11). There have been very few studies done in lower income settings to compare with. However, a longitudinal 16S-based study of 21 refugee infants on the Thailand-Myanmar border found that colonization was dominated by five taxa (*Moraxella*, *Streptococcus*, *Haemophilus*, *Corynebacterium*, and “*Candidatus* Ornithobacterium hominis” [37]), with 15 OTUs accounting for 98.6% of the microbiota. In this cohort, there was development of the microbiota over time but relatively less inter-individual variation.

Lineage-level data are of critical importance for tracking bacterial outbreaks (42), vaccine impact (43), and AMR (44, 45). Traditional colony pick whole genome sequencing (WGS) remains appropriate for analyzing isolates from clinical infection episodes. However, there are limitations using this approach for situations where multiple strains may co-exist such as in the nasopharynx. Multiple colony picks are unlikely to capture full diversity (46), and metagenomic sequencing has become the preferred approach. However, the costs and the low biomass of the nasopharynx makes this challenging. The present study, in agreement with recent findings from the Thailand-Myanmar border which focused entirely on pneumococcal co-colonization (26), confirms the potential for targeted metagenomic sequencing from an initial bacterial culture plate, an approach which resolves both the cost and biomass issues.

There were several limitations to the study. The sample size and follow-up duration were relatively small, limiting the possibilities for definitive analysis of the associations between clinical and environmental factors and the microbiota. Anaerobic culture was not attempted, resulting in an absence of such organisms from the data set, which limited the possibility for detection of inter-species interactions. The selection of a single chocolate agar plate as the enrichment step for mSWEEP work may have also resulted in sub-optimal detection of some aerobic species, notably *S. pneumoniae*, which was more frequently detected by culture and MALDI-TOF where an additional selective blood agar plate culture was included to ensure pneumococcal colonies were identifiable for serotyping. The enrichment culture step resulted in an absence of absolute abundances for the targeted metagenomic sequencing data, limiting the granularity of analyses of inter-species interactions and temporal colonization dynamics. The MALDI-TOF MS identification results were not confirmed directly by conventional or molecular microbiology, except for *S. pneumoniae*, which prevented comment on the overall accuracy of MALDI-TOF MS for identification of upper respiratory organisms. This is of relevance given the plethora of closely related streptococcal species which have been a challenge to identify definitively, including by MALDI-TOF MS (47, 48). Addition of colony pick WGS data would have been valuable. Finally, the mSWEEP database was incomplete, resulting in comparisons between MALDI-TOF MS and mSWEEP being limited to 21 species. Additionally, the small number of reference genomes available for *H. influenzae* and *M. catarrhalis* made it impossible to accurately resolve strain-level carriage dynamics, which was also true to a lesser degree for *S. aureus.* Moving forward, demix_check, a recently described add-on tool to the mSWEEP and mGems pipelines will help with improving removal of spurious multiple colonization detection occurring because of the lack of suitable references. Ongoing efforts to sequence large collections of *H. influenzae* and *M. catarrhalis* carriage isolates will also improve future database coverage and thus strain-level identification. Despite these limitations, the study has demonstrated the value of this analytic approach for the study of the dominant and disease-associated members of the nasopharyngeal microbiota. The culture + MALDI-TOF component provided an assessment of the breadth of colonization while the culture + mSWEEP work assessed intra-species diversity. Future studies should select the workflow component appropriate for the scientific question to be addressed.

### Conclusions

Culture of nasopharyngeal swabs followed by MALDI-TOF MS and targeted metagenomic sequencing was an effective method to determine major components of the bacterial microbiota and within-species diversity. Used at scale, this approach will be useful for determination of impacts on the bacterial microbiota of environmental factors and clinical interventions, such as antibiotics and vaccines.

## Data Availability

The data sets used and/or analyzed during the current study are available from the Mahidol-Oxford Tropical Medicine Research Unit Data Access Committee on reasonable request (https://www.tropmedres.ac/units/moru-bangkok/bioethics-engagement/data-sharing). Targeted metagenomic sequence data for the 620 swabs are available at the European Nucleotide Archive (ENA) under project PRJEB30246/ERP112678.

## SUPPLEMENTAL MATERIAL

Fig. S8 mbio.00784-24-s0001.pdf Hierarchical clustering analysis of culture + MALDI-TOF MS data for 618 nasopharyngeal swabs.

Supplemental material mbio.00784-24-s0002.docx Supplemental figures and Tables S3 to S6.

Table S1 mbio.00784-24-s0003.xlsx Sequencing read accession numbers for the 620 swabs included in the mSWEEP analyses.

Table S2 mbio.00784-24-s0004.xlsx mSWEEP reference database details.

ASM does not own the copyrights to Supplemental Material that may be linked to, or accessed through, an article. The authors have granted ASM a non-exclusive, world-wide license to publish the Supplemental Material files. Please contact the corresponding author directly for reuse.
