# Supplementary figures and images for: Exploring the pediatric nasopharyngeal bacterial microbiota with culture-based MALDI-TOF mass spectrometry and targeted metagenomic sequencing
Source: mBio. 2024 Apr 29;15(6):e00784-24. doi: 10.1128/mbio.00784-24 (PMC11237702; doi:10.1128/mbio.00784-24)

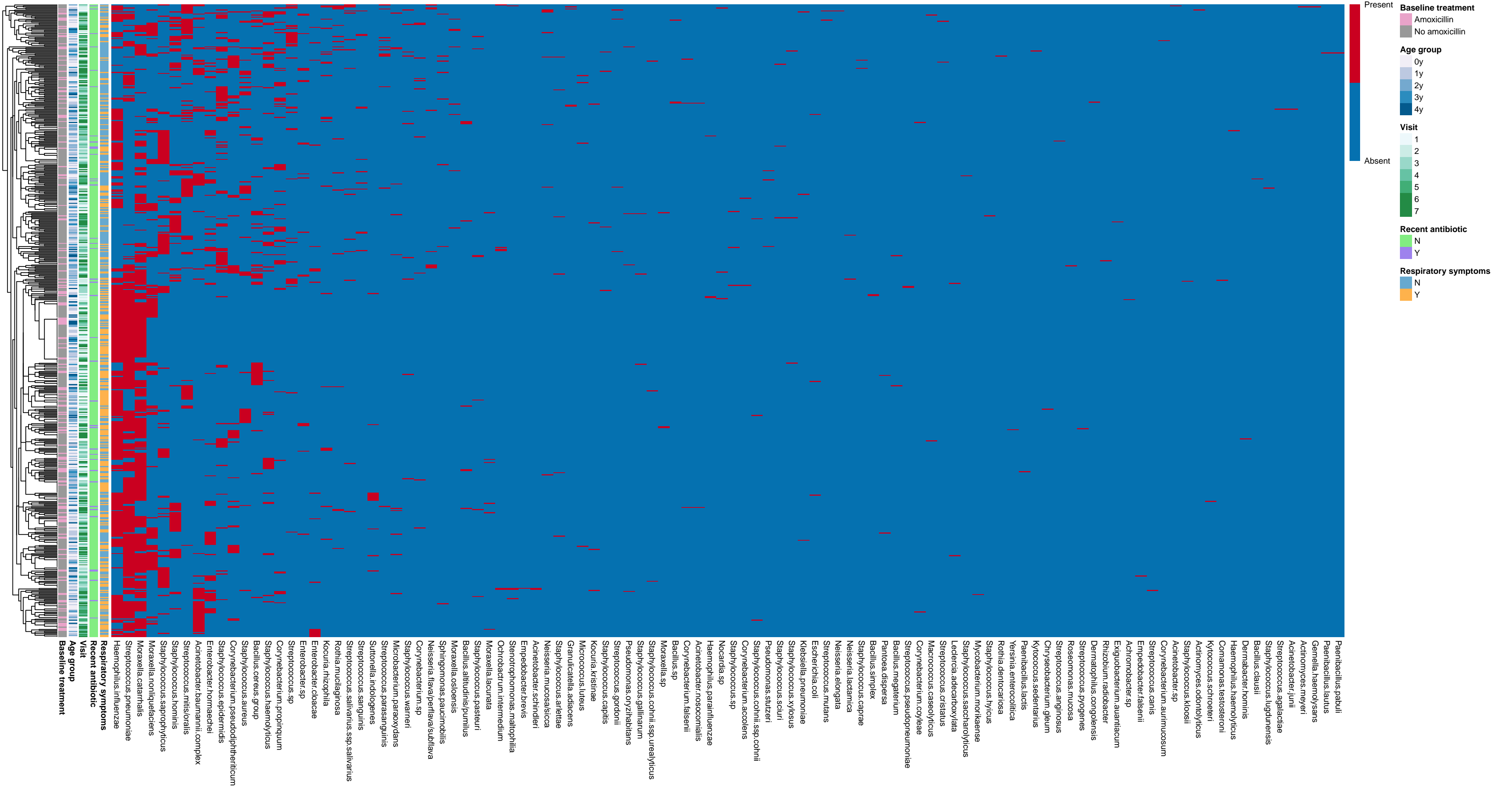

Supplement: Fig. S8 — Hierarchical clustering analysis of culture + MALDI-TOF MS data for 618 nasopharyngeal swabs. [file mbio.00784-24-s0001.pdf]
