# Supplementary material for: Exploring the pediatric nasopharyngeal bacterial microbiota with culture-based MALDI-TOF mass spectrometry and targeted metagenomic sequencing
Source: mBio. 2024 Apr 29;15(6):e00784-24. doi: 10.1128/mbio.00784-24 (PMC11237702; doi:10.1128/mbio.00784-24)
Supplement: Supplemental material — Supplemental figures and Tables S3 to S6. [file mbio.00784-24-s0002.docx]

**Supplementary Material**

Exploring the paediatric nasopharyngeal bacterial microbiota with culture-based MALDI-TOF mass spectrometry and targeted metagenomic sequencing

Sreymom Pol, Teemu Kallonen, Tommi Mäklin, Poda Sar, Jill Hopkins, Sona Soeng, Thyl Miliya, Clare L. Ling, Stephen D Bentley, Jukka Corander, Paul Turner

**Supplementary Table 1 (Excel file). Sequencing read accession numbers for the 620 swabs included in the mSWEEP analyses.**

**Supplementary Table 2 (Excel file). mSWEEP reference database details.**


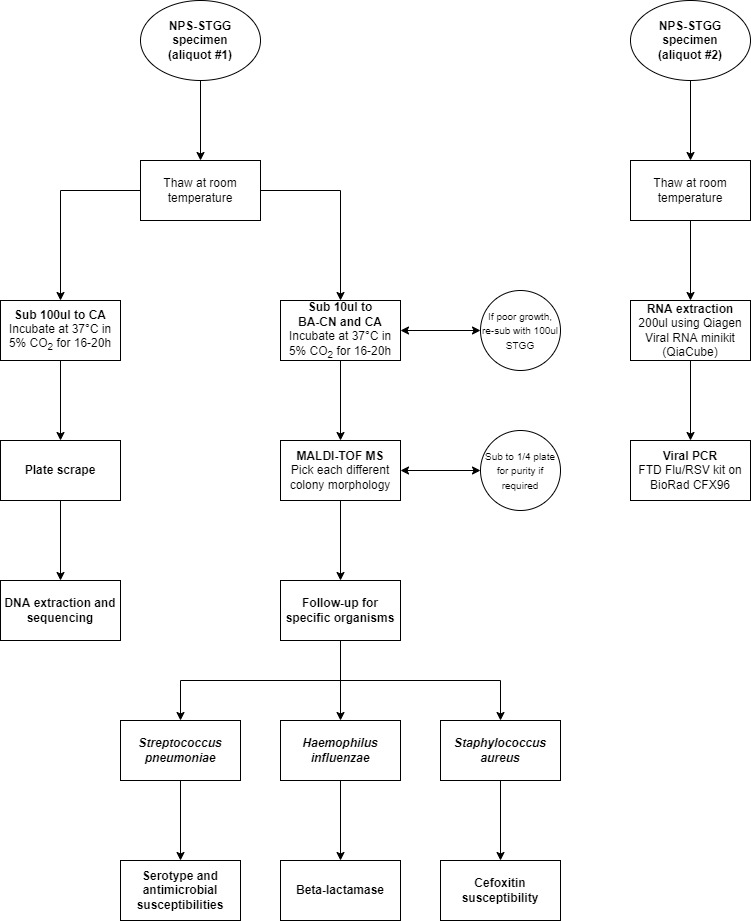


**Supplementary Figure 1**. **Nasopharyngeal swab processing summary**

NPS-STGG: nasopharyngeal swab – skim milk-tryptone-glucose-glycerol broth specimen; CA: chocolate agar; BA-CN: sheep blood – gentamicin agar; Flu: influenza A / B; RSV: respiratory syncitial virus.

| **Serotype** | **Vaccine type*** | **Count** |
| --- | --- | --- |
| 23A | NVT | 43 |
| 6B | PCV13 | 40 |
| 15B/C^†^ | NVT | 33 |
| 6A | PCV13 | 22 |
| 34 | NVT | 19 |
| 11A | NVT | 18 |
| 15A | NVT | 16 |
| 19F | PCV13 | 16 |
| 18C | PCV13 | 15 |
| NT | NT | 14 |
| 35B | NVT | 13 |
| 6C | NVT | 13 |
| 14 | PCV13 | 11 |
| 19A | PCV13 | 11 |
| 23F | PCV13 | 10 |
| 38 | NVT | 7 |
| 33B | NVT | 6 |
| 17F | NVT | 5 |
| 23B | NVT | 5 |
| 3 | PCV13 | 5 |
| 13 | NVT | 4 |
| 24F | NVT | 3 |
| 28F | NVT | 3 |
| 9A | NVT | 3 |
| 10A | NVT | 2 |
| 19B | NVT | 2 |
| 35C | NVT | 2 |
| 10B | NVT | 1 |
| 10F | NVT | 1 |
| 16F | NVT | 1 |
| 18F | NVT | 1 |

*PCV13: covered by 13-valent pneumococcal conjugate vaccine; NVT: non-vaccine serotype; NT: non-typeable

^†^Combined for clarity (15B: 21 isolates; 15C: 12 isolates)

**Supplementary Table 3. *Streptococcus pneumoniae* serotypes identified from 620 nasopharyngeal swab cultures**

**
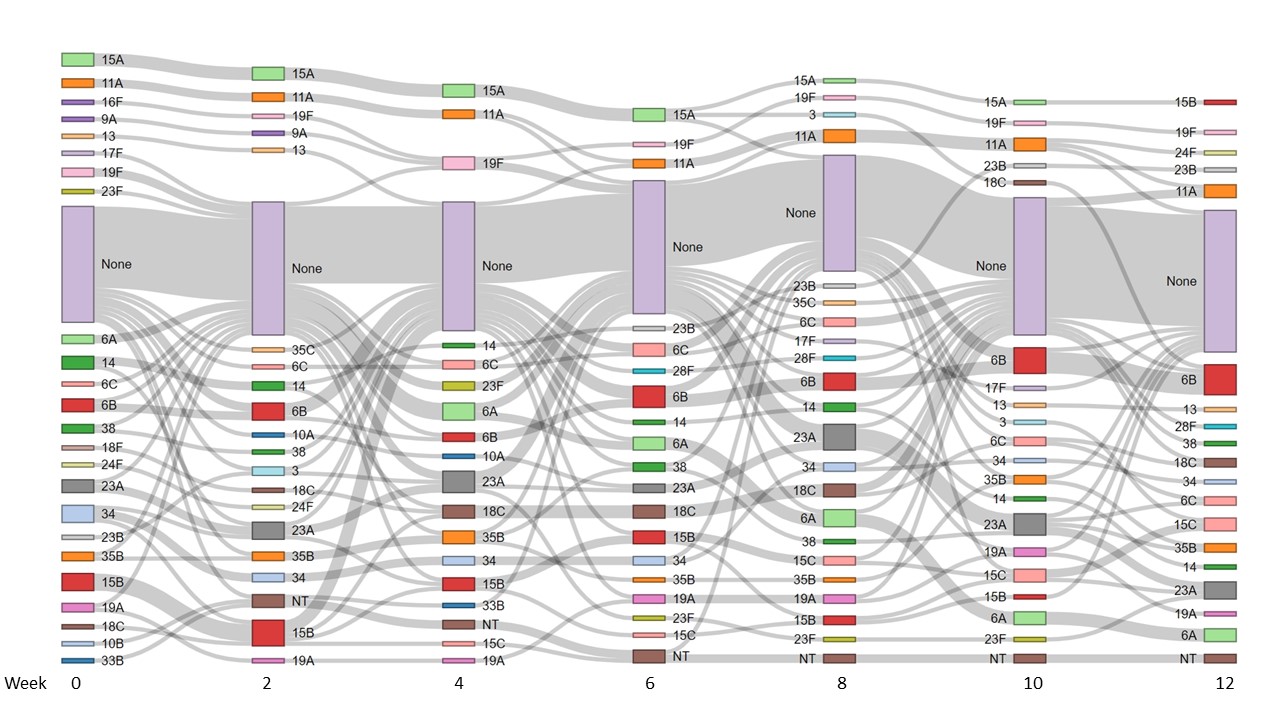
**

**Supplementary Figure 2. Pneumococcal serotype detection from nasopharyngeal swab culture, by visit.**

Each individual is represented by a grey line, moving from left to right along the visits (week 0 to week 12), with pneumococcal serotypes detected at each visit represented by coloured rectangles. Only the 71 children with complete swab sets are included in this plot.


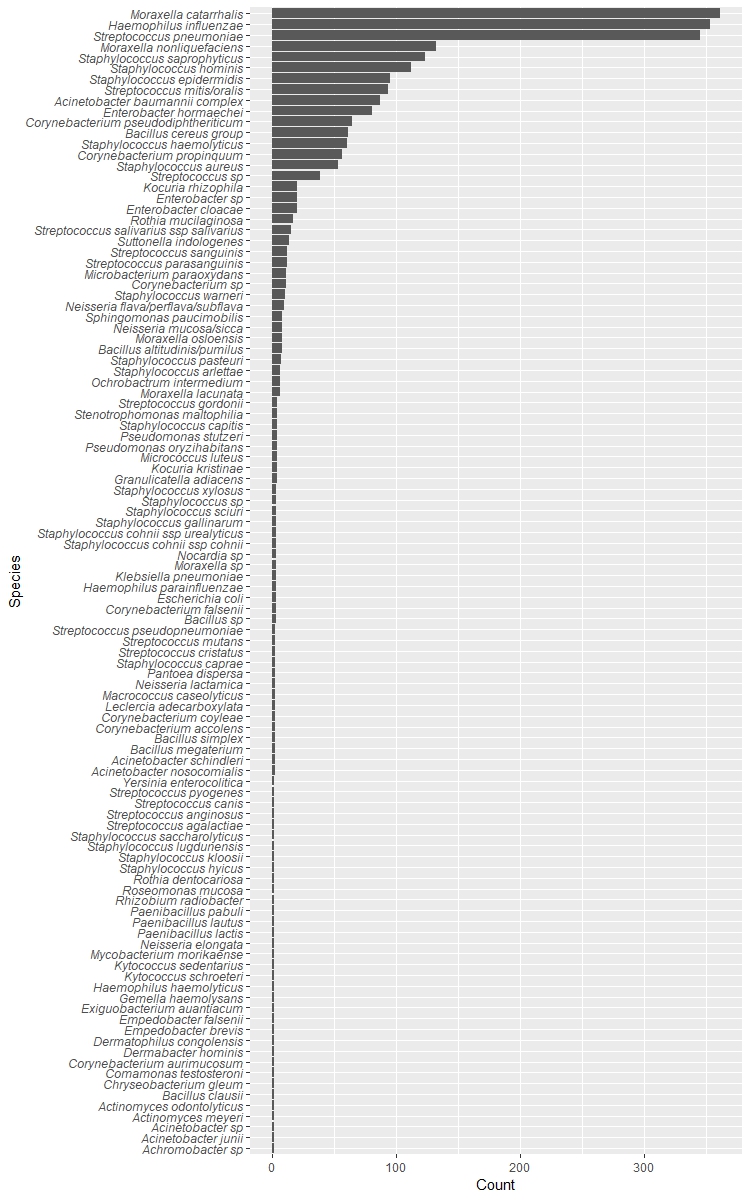


**Supplementary Figure 3. Bacterial species detected by MALDI-TOF MS of morphologically distinct colonies from chocolate agar culture plates of 620 nasopharyngeal swabs**

**
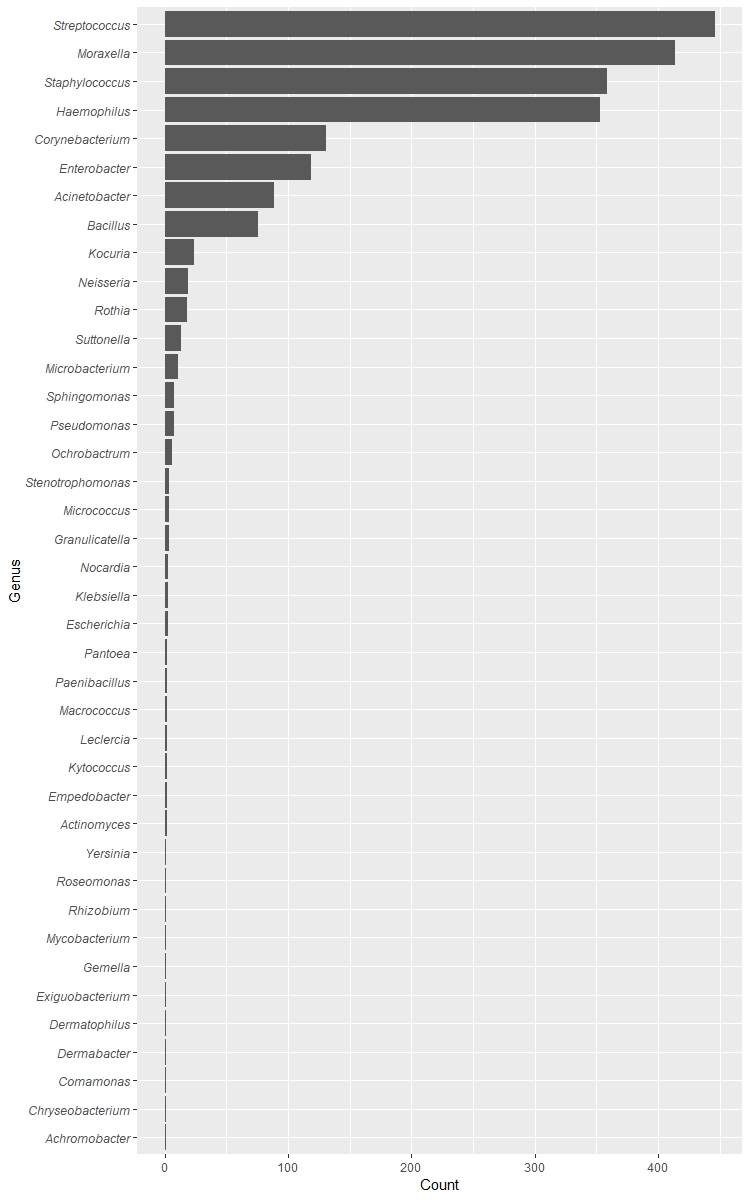
**

**Supplementary Figure 4. Bacterial genera detected by MALDI-TOF MS of morphologically distinct colonies from chocolate agar culture plates of 620 nasopharyngeal swabs**


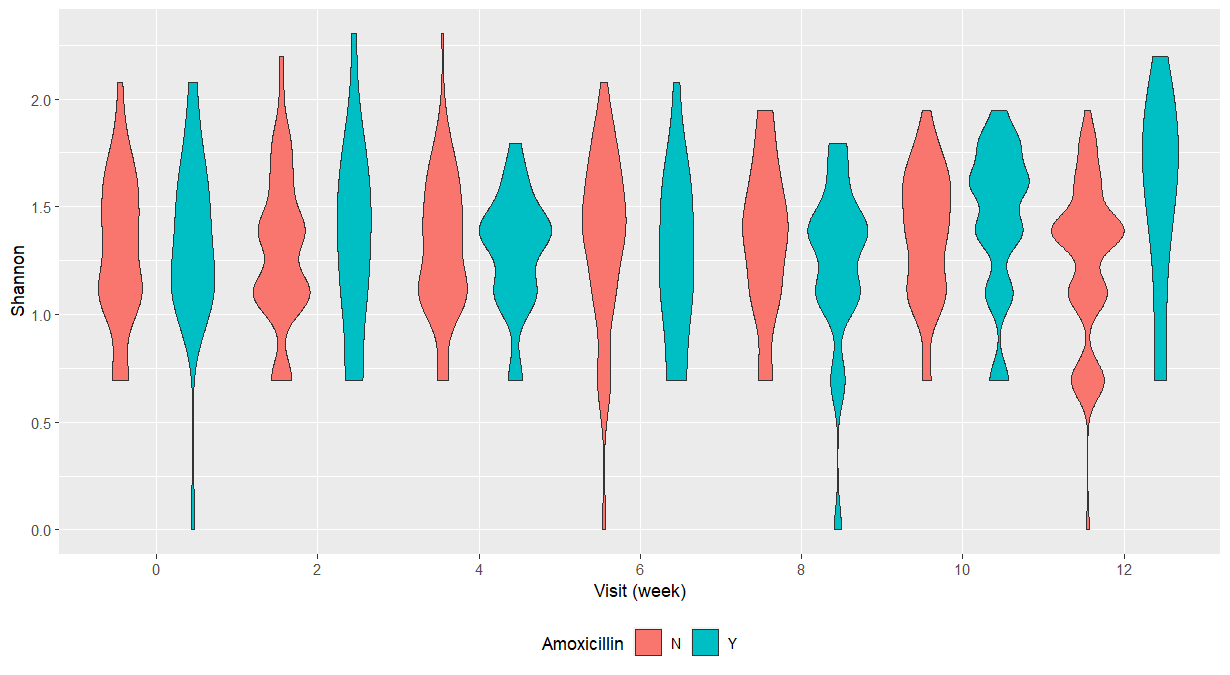


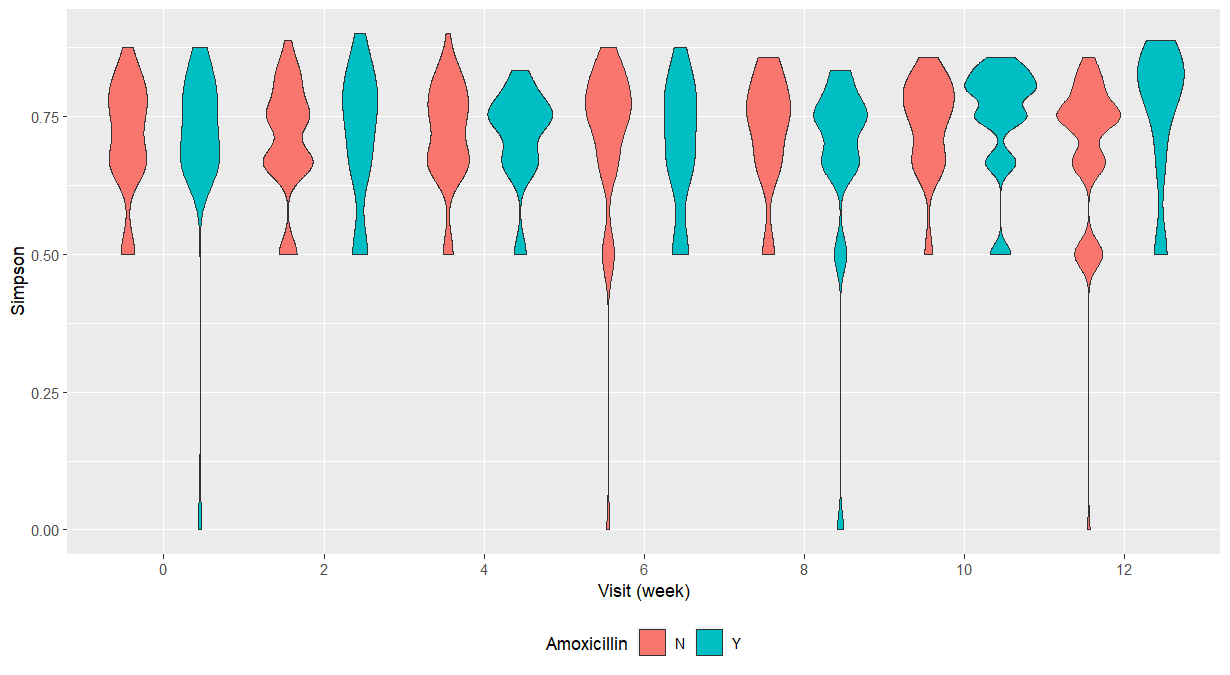


**Supplementary Figure 5. Violin plots of alpha diversity over time in 618 nasopharyngeal swab cultures collected from 101 children at 7 study visits over a 12-week period, stratified by receipt of amoxicillin at the baseline outpatient visit**

The top panel summarises the Shannon diversity index and the bottom panel the Simpson diversity index. Two outliers were removed from the original dataset of 620 swabs.


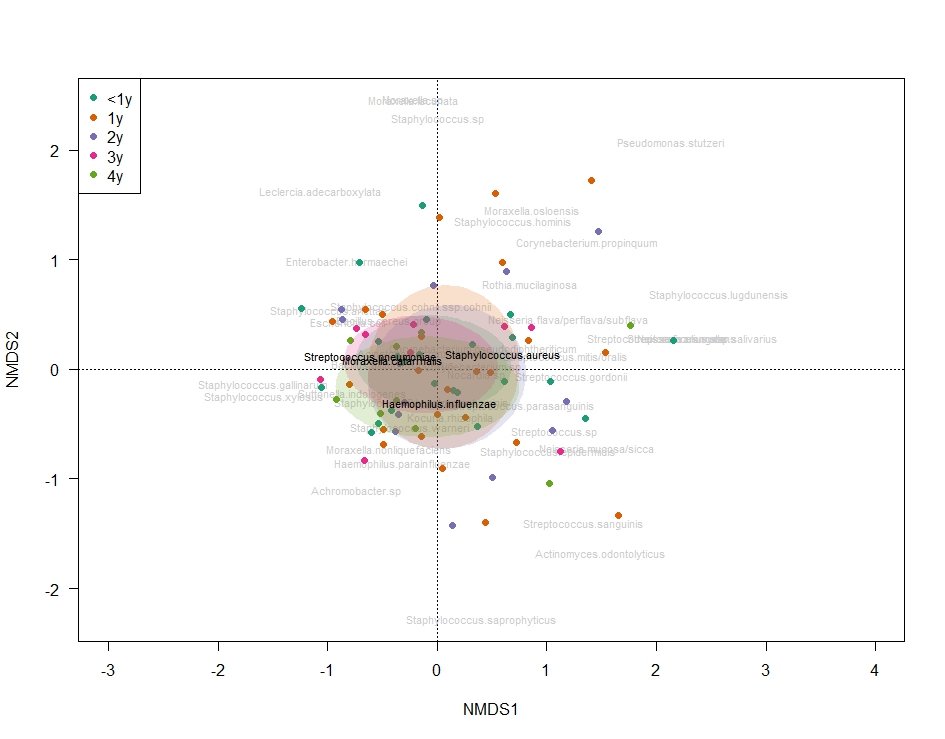


**Supplementary Figure 6. Non-metric multidimensional scaling (NMDS) plot based on Jaccard distance from 101 baseline visit nasopharyngeal swabs, by age group**


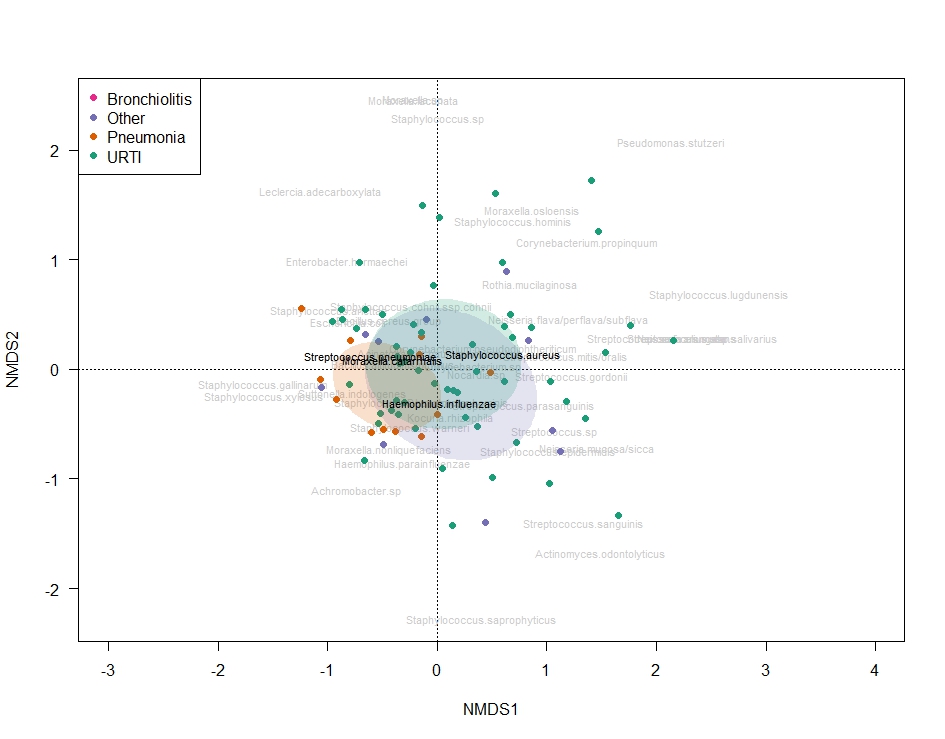


**Supplementary Figure 7. Non-metric multidimensional scaling (NMDS) plot based on Jaccard distance from 101 baseline visit nasopharyngeal swabs, by clinical diagnosis**

| **Variable** | **Univariable model** | | **Multivariable model** | |
| --- | --- | --- | --- | --- |
|  | **R^2^** | **p-value** | **R^2^** | **p-value** |
| Age group | 0.03821 | 0.536 | 0.03821 | 0.600 |
| Household size | 0.00718 | 0.717 | 0.00752 | 0.708 |
| PCV13* (>=2 doses) | 0.01639 | 0.086 | 0.01464 | 0.166 |
| Hib^†^ vaccine (>=2 doses) | 0.00667 | 0.767 | 0.00649 | 0.808 |
| Breast fed | 0.00680 | 0.760 | 0.01047 | 0.382 |
| Household smoker | 0.00283 | 0.998 | 0.00465 | 0.941 |
| Day care attendance | 0.00854 | 0.565 | 0.00588 | 0.852 |
| Current respiratory symptoms | 0.01900 | 0.036 | 0.01287 | 0.239 |
| Febrile (>37.5°C) | 0.00993 | 0.444 | 0.01143 | 0.346 |
| Baseline diagnosis | 0.02784 | 0.549 | 0.01980 | 0.919 |
| Amoxicillin prescription | 0.00701 | 0.742 | 0.00832 | 0.624 |

*13-valent pneumococcal conjugate vaccine; ^†^*Haemophilus influenzae* type b vaccine

**Supplementary Table 4. Results of PERMANOVA tests of 101 baseline swab Jaccard distances against clinical / environmental characteristics**

**Supplementary Figure 8 (PDF). Hierarchical clustering analysis of culture + MALDI-TOF MS data for 618 nasopharyngeal swabs**

Each row indicates an individual swab sample, with species represented by columns (red = present, blue = absent). Two outliers were removed from the original dataset of 620 swabs: one with no bacterial growth and another which grew only *Corynebacterium* sp. and *Pseudomonas stutzeri*, which resulted in failure of model convergence.

|  |  | **Detected by culture + mSWEEP** | |  |
| --- | --- | --- | --- | --- |
|  |  | **Yes** | **No** | **Total** |
| **Detected by culture + MALDI-TOF MS** | **Yes** | 11,009 (84.6) | 347 (2.7) | 11,356 |
|  | **No** | 549 (4.2) | 1,115 (8.6) | 1,664 |
|  | **Total** | 11,558 | 1,462 | 13,020 |

**Supplementary Table 5. Species detection by culture + MALDI-TOF MS versus culture + mSWEEP from nasopharyngeal swab specimens**

Species presence by mSWEEP was defined as 1% relative abundance. Comparisons were restricted to the bacterial species contained in the mSWEEP database, thus each swab contributed 21 observations.


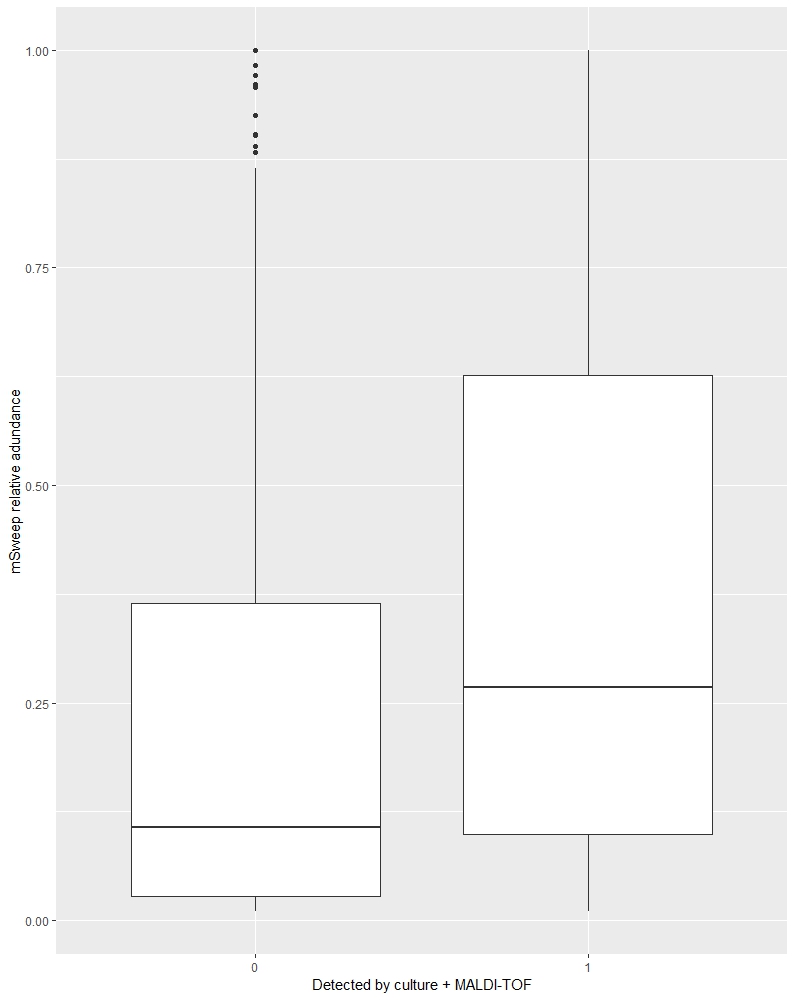


**Supplementary Figure 9. mSweep relative abundance versus culture + MALDI-TOF MS detection for species that were detected by culture + mSweep**

0: not detected by culture + MALDI-TOF MS; 1: detected by culture + MALDI-TOF MS.

| ***S. pneumoniae* lineage** | **Count (%)** |
| --- | --- |
| BAPS cluster 1 | 83 (12%) |
| BAPS cluster 7 | 65 (9.2%) |
| BAPS cluster 11 | 62 (8.8%) |
| BAPS cluster 31 | 46 (6.5%) |
| BAPS cluster 2 | 41 (5.8%) |
| BAPS cluster 12 | 41 (5.8%) |
| BAPS cluster 14 | 40 (5.7%) |
| BAPS cluster 9 | 35 (5.0%) |
| BAPS cluster 21 | 35 (5.0%) |
| BAPS cluster 22 | 33 (4.7%) |
| BAPS cluster 26 | 32 (4.5%) |
| BAPS cluster 18 | 30 (4.2%) |
| BAPS cluster 5 | 26 (3.7%) |
| BAPS cluster 16 | 22 (3.1%) |
| BAPS cluster 27 | 19 (2.7%) |
| BAPS cluster 20 | 18 (2.5%) |
| BAPS cluster 25 | 17 (2.4%) |
| BAPS cluster 3 | 14 (2.0%) |
| BAPS cluster 17 | 12 (1.7%) |
| BAPS cluster 30 | 10 (1.4%) |
| BAPS cluster 6 | 7 (1.0%) |
| BAPS cluster 13 | 5 (0.7%) |
| BAPS cluster 28 | 5 (0.7%) |
| BAPS cluster 8 | 3 (0.4%) |
| BAPS cluster 32 | 2 (0.3%) |
| BAPS cluster 4 | 1 (0.1%) |
| BAPS cluster 10 | 1 (0.1%) |
| BAPS cluster 24 | 1 (0.1%) |

**Supplementary Table 6. *Streptococcus pneumoniae* lineages detected from 620 nasopharyngeal swab cultures using the mSweep pipeline**

Detection was defined as an overall relative abundance of ≥0.01.


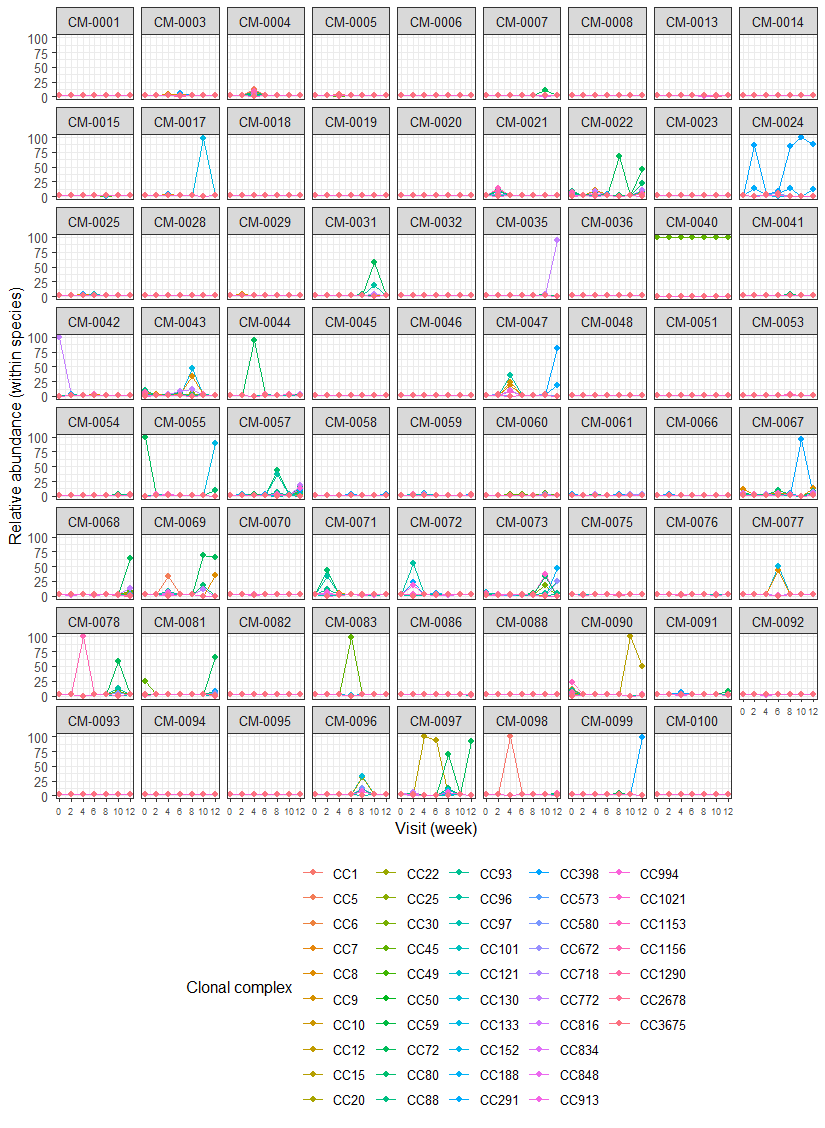


**Supplementary Figure 10.** **Dynamics of *Staphylococcus aureus* colonisation over time, in 71 children with complete nasopharyngeal swab sets**

Each child is documented in an individual plot, with the within-species relative abundance of each *S. aureus* clonal complex plotted as a separate colour. Clonal complexes (CC) were defined by double-locus variants of the central multi-locus sequence type (ST).
